# Reply to Rugge, M. Autoimmune Gastritis Diagnosis: Encompassing Pessimism, Realism, and Wish for the Future. Comment on “Vienneau et al. Autoimmune Metaplastic Atrophic Gastritis Reporting: Are Pathologists and Endoscopists on the Same Page? *Diagnostics* 2025, *15*, 2906”

**DOI:** 10.3390/diagnostics16071074

**Published:** 2026-04-02

**Authors:** Nicole Vienneau, Hwajeong Lee, Micheal Tadros

**Affiliations:** 1 Department of Pathology and Laboratory Medicine, Albany Medical Center, 43 New Scotland Avenue, Albany, NY 12208, USA; 2 Division of Gastroenterology, Department of Medicine, Albany Medical Center, 43 New Scotland Avenue, Albany, NY 12208, USA

Thank you for your thoughtful and insightful commentary on our manuscript regarding autoimmune metaplastic atrophic gastritis (AMAG) [1]. We greatly appreciate your careful reading, your recognition of the rigorous re-evaluation process, and your endorsement of the core conclusions emphasizing enhanced adherence to biopsy guidelines, standardized pathology reporting, and consistent surveillance for AMAG patients.

We fully agree that the cultural and operational challenges you describe represent significant barriers to the optimal management of AMAG. The reluctance to follow established biopsy protocols is indeed widespread, often driven by cost pressures, workload concerns, differing practices depending on which pathology laboratory the samples will be sent to, and limited interdisciplinary communication. As you rightly point out, the frequent absence of essential clinical context may leave both endoscopists and pathologists operating in isolation from the pertinent clinical questions, potentially contributing to the under-recognition of AMAG and delayed diagnoses.

We also share your concern about the persistence of minimalistic or purely descriptive pathology reports, even in the face of international guidelines and supporting literature. This approach, while protective against over-interpretation in the absence of clinical context, may not meet the expectations of clinicians and patients who would benefit from actionable insights. Greater standardization could address this gap, perhaps through education and reporting templates.

Regarding surveillance, your comments on the evolving understanding of cancer risk in AMAG are particularly valuable. While recent evidence supports a low risk of gastric adenocarcinoma in *Helicobacter pylori*-naive AMAG patients [2], the literature appears to remain mixed, with some meta-analyses suggesting elevated risk of gastric cancer perhaps depending on AMAG stage [3]. These studies, as you point out, may be influenced by the inclusion of cases with mixed etiologies, including undetected *Helicobacter pylori* exposure. Current MAPS III guidelines suggest that patients with AMAG should have high quality endoscopic follow-up every 3 years to detect neuroendocrine tumors and gastric cancer [4]. Further, they also specify that individuals with advanced stages of atrophy or intestinal metaplasia are followed with high-quality endoscopy every 3 years [4]. This aligns with individualized, etiology-specific approaches to distinguish AMAG alone from comorbid cases, while emphasizing the importance of high-quality endoscopy [5].

Lastly, your proposal that artificial intelligence (AI)-driven clinical networking could help bridge the gaps in interdisciplinary communication is intriguing and timely. Emerging AI applications in endoscopy, pathology, and integrated data platforms (for sharing clinical, serological, and histological information) may hold promise for fostering collaborative, patient-centered care [5,6]. Such tools could certainly be a valuable avenue for further exploration in the near future.

Once again, thank you for this enriching commentary, which highlights important practical and conceptual challenges in the field. We believe it will stimulate valuable communication and contribute to improved standards of care for patients with AMAG.

## Abbreviations

The following abbreviations are used in this manuscript:

AMAG	Autoimmune metaplastic atrophic gastritis
MAPS	Management of precancerous conditions and lesions in the stomach
AI	Artificial intelligence
